# Assessment of the root surface temperature during the use of intracanal agitation systems: In vitro study

**DOI:** 10.1590/0103-6440202305517

**Published:** 2023-10-27

**Authors:** Barbara S. Fontanezi, Juliana D. Bronzato, Nelson T. Mohara, Adriana de-Jesus-Soares, Marcos Frozoni

**Affiliations:** 1 Department of Endodontics, São Leopoldo Mandic School of Dentistry, Campinas, SP, Brazil.; 2 Department of Restorative Dentistry, Division of Endodontics, Piracicaba Dental School, State University of Campinas, Piracicaba, SP, Brazil.

**Keywords:** endodontics, ultrasonics, temperature, root canal irrigants

## Abstract

This study aimed to evaluate the temperature changes that the different methods of agitation of irrigants promote on the external dental root surface. Nine extracted human lower premolars were standardized by cone-beam computed tomography and used. The root canal was instrumented with a Reciproc 40.06 file. Temperature measurement was performed using K-type thermocouple sensors attached to the middle, cervical, and apical thirds of the teeth. The teeth had their roots immersed in distilled water at 37ºC, which were distributed into 3 experimental groups according to the mechanical agitation methods to be studied. US Group (n=3), Irrisonic Ultrasonic Tip activated through ultrasound; EC Group (n=3), Easyclean Tip coupled to a contra-angle low-speed handpiece; XP Group (n=3), XP-endo Finisher file coupled to an endodontic electrical motor. Temperature measurements were performed simultaneously with agitation and irrigation of intracanal irrigants. Statistical analysis was performed using SPSS software with a significance level of 5%. For multiple comparisons, the Tukey test was used. The association between mechanical agitation methods and root third was statistically significant. Regarding the temperatures recorded on the external surface of the roots, the ultrasonic tip was significantly higher than the XP-endo Finisher file and the Easyclean tip, which did not differ from each other. Regarding the ultrasonic tip, the external temperature in the middle third (39.46ºC) of the root was significantly lower than in the cervical (40.41ºC) and apical third (40.53ºC). None of the agitation methods of irrigants studied presented heating above 47ºC, and their use is safe for periodontal tissues.

## Resumo

Este estudo teve como objetivo avaliar as mudanças de temperatura que os diferentes métodos de agitação de irrigantes promovem na superfície externa da raiz dentária. Nove pré-molares humanos extraídos foram padronizados por tomografia computadorizada de feixe cônico e usados. O canal radicular foi instrumentado com uma lima Reciproc 40.06 de 25 mm. A medição da temperatura foi realizada usando sensores termopares do tipo K fixados nos terços médio, cervical e apical dos dentes. Os dentes tiveram suas raízes imersas em água destilada a 37ºC, as quais foram distribuídas em 3 grupos experimentais de acordo com os métodos de agitação mecânica a serem estudados. Grupo US (n=3), Ponta Irrisonic Ultrasonic ativado por ultrassom; Grupo EC (n=3), Ponta Easyclean acoplada a um contra-ângulo de baixa rotação; Grupo XP (n=3), Lima XP-endo Finisher acoplada a um motor elétrico endodôntico. As medições de temperatura foram realizadas simultaneamente com agitação e irrigação de irrigantes intracanal. A análise estatística foi realizada no software SPSS com nível de significância de 5%. Para comparações múltiplas, foi utilizado o teste de Tukey. A associação entre métodos de agitação mecânica e terço radicular foi estatisticamente significativa. Em relação às temperaturas registradas na superfície externa das raízes, a ponta ultrassônica foi significativamente maior que a lima XP-endo Finisher e a ponta Easyclean, que não diferiram entre si. Com relação à ponta ultrassônica, a temperatura externa no terço médio (39,46ºC) da raiz foi significativamente menor do que nos terços cervical (40,41ºC) e apical (40,53ºC). Nenhum dos métodos de agitação dos irrigantes estudados (Irrisonic, XP-endo Finisher e Easyclean) apresentou aquecimento acima de 47ºC, sendo seu uso seguro para os tecidos periodontais.

## Introduction

The activation of irrigants is considered a highly effective way to improve intracanal disinfection ^1^ since manual and mechanical instruments cannot touch all canal walls ^2^. To overcome these obstacles, instruments with different designs and concepts have been developed over the years to promote greater effectiveness of irrigating solutions, enhance disinfection, and increase the predictability and success of endodontic treatment, especially in cases where there is anatomic complexity ^3^ ^,^ ^4^.

Passive ultrasonic irrigation (PUI) consists of the placement of the irrigating solution inside the root canal, and then its activation passively so that the instrument used for activation does not touch the canal walls ^5^. Activation using ultrasonic tips is a technique widely cited in the literature ^6^ ^,^ ^7^. The Irrisonic tip (20.01, Helse Dental Technology, Santa Rosa de Viterbo, Brazil) is recommended for performing this technique.

Different instruments can be used for the agitation of irrigant solutions inside the canal. The XP-endo Finisher (25.00, FKG, La Chaux-de-Fonds, Switzerland) is a finishing rotary file and it was developed to improve disinfection and cleaning after root canal shaping ^8^. It is made of MaxWire nickel-titanium alloy (Martensite-Austenite, EletropolishFlex, FKG), which can deform according to the intracanal temperature, touching the walls and promoting the agitation of the irrigating solution ^9^. Easyclean (25.04, Easy Equipaments, Belo Horizonte, MG, Brazil) is a flexible plastic instrument ^6^ that can be used in reciprocating or rotary kinematics. However, rotary motion has been shown to be more effective in removing debris ^10^.

There is a wide variety of protocols associated with irrigant agitation. Sodium hypochlorite (NaOCI) is the most used irrigant in endodontic practice, mainly due to its ability to dissolve organic tissue and antimicrobial properties ^11^ ^,^ ^12^. Several NaOCI concentrations from 0.5% to 6% ^13^ can be found in the endodontic literature. The sonic or ultrasonic activation of NaOCI promotes, in addition to cavitation, heating of the irrigating solution ^13^ that causes an acceleration in the collagen dissolution rate ^12^ ^,^ ^14^ ^,^ ^5^.

Hence, there is a concern about root surface temperature change and heat transfer to periodontal tissues. According to some studies, the increase in temperature above 10ºC in relation to body temperature - 37ºC ^16^ ^,^ ^17^ ^,^ ^8^, can cause irreparable injuries to periodontal tissues as blood flow interruption and necrosis ^16^. Previous research has linked the use of ultrasonic inserts to an increase in temperature during irrigant activation per the manufacturer's recommendations ^16^ ^,^ ^20^ ^,^ ^21^.

There is a limitation in the literature associating new methods of irrigant agitation and the temperature they can cause on the external root surface when activated. The present study aims to evaluate the temperature changes on the external dental root surface during the use of different methods of irrigant agitation. The null hypothesis tested is that there will be no difference in the temperature of the external root surface subjected to different methods of irrigant agitation.

## Materials and methods

**Figure 1:**
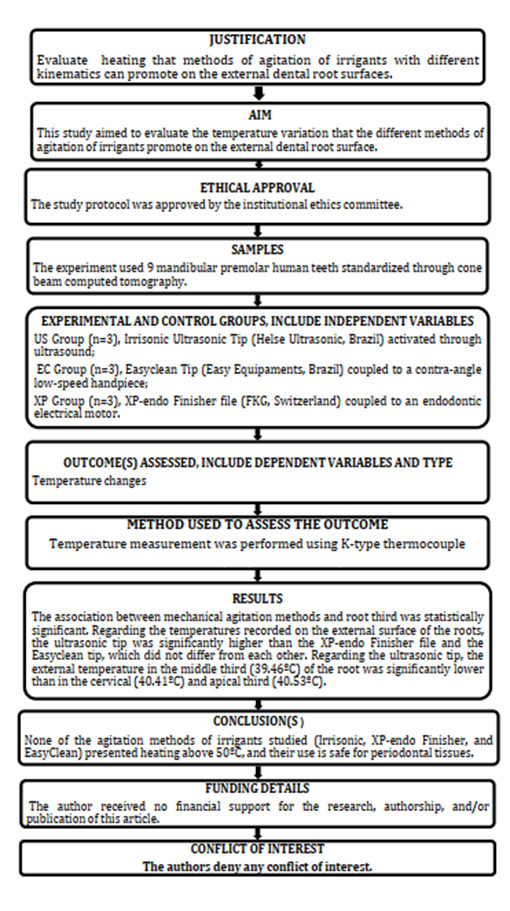
PRILE flowchart

The study was conducted following PRILE guidelines for reporting laboratory study ^22^ (Figure 1).

### Sample size calculation

To perform the sample size calculation, the program G*Power3.1.9.4 was used, considering the analysis of variance for repeated measures. The effect size of 0.78 was obtained considering the N from a previous study ^18^, with a significance level of 5%, and a power of 90%, the sample size calculation indicated the need for at least three teeth per group in the present study (n = 3).

### Sample selection

The samples were obtained through the donation of patients undergoing tooth extraction for orthodontic or periodontal reasons. The patients were informed about the research by an informed consent form. Thus, the biorepository was authorized by the São Leopoldo Mandic-Campinas\SP Dental Research Center (process number 43860921.0.0000.5374). The teeth were disinfected in 5% NaOCI for 1 hour and stored in saline solution at room temperature until use.

Teeth with a single canal and foramen, absence of internal and external resorption, calcifications, cracks, and fractures were included in the study. The teeth had a root length of 15 mm from the dental apex to the cementoenamel junction. The degree of curvature of the roots was a maximum of 10º, characterizing a slight curvature according to the Schneider classification ^23^.

In the first step, the tooth access cavity preparation was done, and the selected samples had a foramen compatible with a NiTi #25.02 hand file (Dentsply Maillefer, Ballaigues, Switzerland) introduced inside the root canal until its tip reached the apical foramen, which was observed using a microscope (Alliance, São Carlos, São Paulo, Brazil). Only samples in which a #25.02 NiTi hand file (Dentsply Maillefer, Ballaigues, Switzerland) fit the foramen and a #30.02 NiTi hand file (Dentsply Maillefer, Ballaigues, Switzerland) did not reach the foramen were included in the study.

Twenty-eight samples were initially scanned in cone beam computed tomography (CBCT) (Kavo OP 3D, São Paulo, Brazil) before preparations to obtain a general sketch of the root canal anatomy and allow a homogeneous sample selection. Each sample was positioned on the platform with the roots facing upward. The parameters used were the following: voltage of 95 KV, current of 2.2 µA with resolution and rotation of 360º, 426 mGycm², Endo resolution, and exposure of 20 seconds.

**Figure 2:**
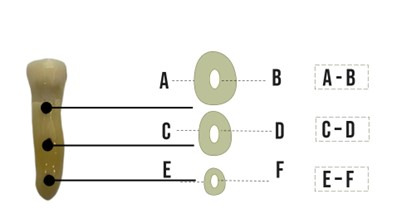
The axial dentin area of the tomographic volumes of the cervical (12 mm from the apex), medium (7 mm from the apex), and apical (2 mm from the apex) thirds. A) Measurement of the dentin plus root canal of the axial section referring to the cervical third. B) Measurement of the root canal referring to the cervical third. The value of A is subtracted from the value of B, resulting in the dentin portion of the sample in the cervical third. C) Measurement of the dentin plus root canal of the axial section, referring to the middle third. D) Measurement of the root canal, referring to the middle third. The value of C is subtracted from the value of D, resulting in the dentin portion of the sample in the middle third. E) Measurement of the dentin plus root canal of the axial section, referring to the apical third. F) Measurement of the root canal, referring to the apical third. The E value is subtracted from the F value, resulting in the dentin portion of the sample in the middle third.

The axial dentin area of the tomographic volumes of the cervical (12 mm from the apex), medium (7mm from the apex), and apical (2mm from the apex) thirds, where the thermocouple sensors would be coupled to the external root surface, was measured with the Radiant software (Medixant, Posnânina, Poland) (Figure 2). Both the total area of the axial slice (containing dentin and root canal) and the area of the root canal were measured. After obtaining these data, the total area was subtracted from the area of the root canal, where it was obtained only the axial dentin area of each dental element in the position where the sensors would be coupled. To prove the homogeneous random distribution of teeth among the three methods, the data collected were submitted to a one-way analysis of variance test (p=0.835). Nineteen samples were excluded.

### Samples preparation

###  Samples standardization 

The procedure was performed by a single operator who is a specialist in endodontics and has 7 years of experience. The samples were measured and standardized at 20 mm in length, which was considered the working length. The teeth had a root length of 15 mm and a crown length of 5 mm. When necessary, the crown was partially cut to achieve the 5 mm measurement.

###  Chemomechanical preparation 

The tooth was inserted into an acrylic tube (RN disposables, Campinas, SP, Brazil) with a capacity of 10 ml, filled with alginate (Zhermack, Badia Polesine, Italy). Only the coronary remnant remained visible to form a closed irrigation model. The irrigating solution used was 2.5% NaOCI (Fórmula e Ação, São Paulo, SP, Brazil). The solution was injected into the root canal with a 21 mm 30G Navitip needle (Ultradent Products Inc, USA) coupled with a 1 ml disposable syringe (INJEX Indústrias Cirúrgicas LTDA, Ourinhos, SP, Brazil), adapted to a peristaltic pump hose (LAP-101-3; MSTCNOPON, Piracicaba, SP, Brazil) at a 5 ml/minute flow rate ^24^.

The root canal was instrumented with a 25 mm Reciproc 40.06 file (VDW, Munich, Germany) with three in-and-out pecking motions driven by the XSmart plus motor (Dentsply Sirona, Ballaigues, Switzerland) in the reciprocating function following the manufacturer's instructions. The pecking motion was repeated until the end of the apical third. The total amount of irrigating solution was 5 ml.

### Preparation of the apparatus for external temperature testing

A glass flask with dimensions of 101 mm in length, 101 mm in width, and 137 mm in height with a plastic cap with a diameter of 88 mm and a height of 23 mm (Invicta, Pouso Alegre, MG, Brazil) was used in the experiment. A hole was created in the center of the plastic cap of the glass vial with a diameter similar to the cervical diameter of the remaining tooth root that was used in the experiment. Marginal sealing was performed with cyanoacrylate glue (Henkel, Düsseldof, Germany), preventing sample displacement during the experiment.

The teeth were inserted into the hole in the plastic cap, up to the limit of the beginning of the cervical third, leaving 15 mm of the root inside the tube and 5 mm of coronary remaining outside the cap. Three holes were also made, lateral to the central hole to fix the thermocouple sensors (Minipa do Brasil, São Paulo, SP, Brazil). The glass flask was filled with 750 ml of distilled water and placed on a hot plate (Solidsteel, Piracicaba, SP, Brazil) that heated the liquid to body temperature at 37°C ^25^ ^,^ ^26^.

###  Sensor positioning 

**Figure 3:**
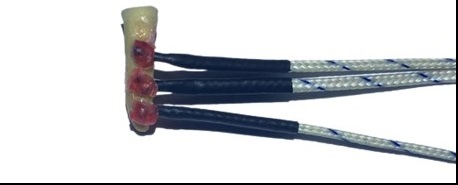
Type K thermocouple sensors fixed in the cervical, middle, and apical thirds of the tooth.

Three K-type thermocouple temperature sensors (Minipa do Brasil) were attached to the cervical, middle, and apical thirds of the tooth root that was inserted into the plastic cap, fixed with a layer of wax (New Wax; Technew, Rio de Janeiro, RJ, Brazil) and wrapped with Nexcare micropore silicone tape (3M, Sumaré, SP, Brazil) in the respective markings (Figure 3):

Sensor T1: Positioned 2mm from the root foramen, representing the apical third.

Sensor T2: Positioned 7mm from the root foramen, representing the middle third

T3 sensor: Positioned 12mm from the root foramen, representing the cervical third.

After positioning the sensors on the dental root, the cap containing the tooth and the fixed sensors was screwed onto the glass bottle with heated distilled water, and the root was immersed in water. The temperature sensors were connected to the 3-channel thermometer (Minipa do Brasil). After reaching the water temperature of 37 °C, the hot plate was turned off and monitored by K-type thermocouple temperature sensors.

### Description of the irrigant agitation technique

The teeth were irrigated with 0.3 ml of 2.5% NaOCI solution (Fórmula e Ação) using a 1ml disposable syringe (Ultradent Products Inc) and a 21 mm Navitip 30G needle (Ultradent Products Inc, USA) with the needle positioned 3 mm short of the working length. The substance was shaken intracanal for 30 seconds with different methods of shaking according to each group ^7^ ^,^ ^9^ ^,^ ^27^. The kinematics chosen followed the manufacturer's recommendation:

US group (n=3): Agitation was performed with an Irrisonic ultrasonic tip with 20% power coupled to a Woodpecker UDSK Ultrasound (Woodpecker, Guilin, China) inserted into the working length and followed by activation for 30 seconds with in-and-out movements with an amplitude of approximately 3 mm, allowing ultrasonic movement.

EC group (n=3): Agitation was performed with Easyclean tips activated for 30 seconds at 15,000 RPM, inserted into the working length, and activated for 30 seconds with in-and-out movements with an amplitude of 3 mm.

XP Group (n=3): Agitation was performed with XP-endo Finisher files. The file was cooled with a refrigerant spray (Maquira, Maringá, SP, Brazil) through the millimeter tube that comes with the file and activated with a torque of 1 N and a speed of 800 RPM as suggested by the manufacturer. The file was inserted into the working length and used for 30 seconds, with slow and smooth longitudinal movements, with penetration and removal of the file each 7 to 8 mm.

Aspiration was then performed using an endodontic suction cannula (Indusbello,

Londrina, PR, Brazil) and new irrigation with 1 ml of NaOCI that lasted 20 seconds. The activation and aspiration/irrigation cycle was repeated three times for each group, following the same time interval for all groups. The total procedure time per sample was 150 seconds and the total volume of the irrigant was 4 ml in all groups.

### Recording of temperature values

An iPhone cell phone camera (Apple Inc, Cupertino, CA, USA) was fixed on a tripod to record the temperature changes obtained by the thermometer (Minipa do Brasil) in the time interval of 2 minutes and thirty seconds, considering the 3 agitation cycles lasting 30 seconds and the 3 pauses for agitation and new irrigation within a period of 20 seconds thus understanding the complete procedure. The images were analyzed and the data was transferred to an Excel table (Microsoft, Albuquerque, USA).

Each of the thirds studied (cervical, middle, and apical) generated 151 temperature values, considering the initial temperature (before the start of the procedure) and the temperature changes in the 150 seconds of filming during the procedure. Thus, each sample generated 453 temperature values, and each group generated 1,359 measurements, considering that each group had 3 samples.

### Statistical analysis

Data normality was checked by the Shapiro-Wilk test. The effect of the mechanical agitation method, the evaluation moment (activation and aspiration/irrigation), and the root third on the external root surface temperature, as well as the triple and double interactions between these three variables under study, were investigated by the three-way analysis of variance for repeated measures. For multiple comparisons, the Tukey test was used. Statistical calculations were performed using SPSS 23 software (SPSS Inc., Chicago, IL, USA), setting the significance level at 5%.

## Results

Table 1 summarizes the temperature values of the external root surface, divided by thirds, at the moments of activation and aspiration/irrigation of each group. There was no statistically significant interaction between the three variables studied, that is, between the methods of mechanical agitation, the evaluation moments (activation and aspiration/irrigation), and the root third (p = 0.933).

**Table 1:** Means and standard deviations of the temperature (^o^C) in the thirds of the root surface, according to the moment of evaluation and the agitation method used.

Agitation method	Activation			Aspiration/Irrigation		
	Cervical	Middle	Apical	Cervical	Middle	Apical
Irrisonic	41.27 (0.37)	42.17 (0.53)	42.48 (0.53)	37.65 (0.15)	38.65 (0.15)	38.59 (0.32)
EasyClean	36.93 (0.27)	37.15 (0.22)	37.03 (0.15)	36.89 (0.26)	37.20 (0.31)	37.03 (0.15)
XP Endo Finisher	36.58 (0.30)	37.01 (0.22)	36.94 (0.18)	36.52 (0.18)	36.94 (0.10)	36.90 (0.18)

Temperature values in the thirds of the root surface, at the moments of activation and aspiration/irrigation with Irrisonic, Easyclean, and the XP Endo Finisher file.

There was also no statistically significant effect of the interaction between the evaluation moments (agitation and aspiration/irrigation) and the root third (p = 0.634). The association between the methods of mechanical agitation and the root third was statistically significant (p =0.004).

Table 2 presents the results of the significant interaction and indicates that the temperatures recorded on the external surface of the roots using the ultrasonic tip were statistically higher than those recorded using the XP-endo Finisher file and Easyclean tip, which did not differ from each other.

Regarding the comparisons between the temperatures observed in the root thirds, considering separately each method of mechanical agitation, it was observed that for the ultrasonic tip, the external temperature in the middle third of the root was significantly lower than in the other thirds (cervical and apical), with no significant temperature difference between them. As for the XP-endo Finisher file and the Easyclean tip, there was no significant difference in the external temperature of the roots when comparing the cervical, middle, and apical thirds with each other.

**Table 2:** Means and standard deviations of the temperature (^o^C) in the thirds of the root surface, according to the agitation method used, disregarding the moment of evaluation (Activation or Aspiration/ Irrigation).

Agitation method	Cervical	Middle	Apical
Irrisonic	40.41 Bb (1.96)	39.46 Ba (2.00)	40.53 Bb (2.17)
Easyclean	37.17 Aa (0.24)	36.91 Aa (0.24)	37.10 Aa (0.16)
XP Endo Finisher	36.98 Aa (0.16)	36.55 Aa (0.22)	36.92 Aa (0.16)

Means followed by distinct capital letters indicate a statistically significant difference between methods regarding the recorded temperature, considering each third separately (comparisons within each column). Means followed by distinct lowercase letters indicate a statistically significant difference in temperature between thirds, considering each method separately (comparisons within each row).

## Discussion

The agitation of chemical substances results in a rise in temperature within the root canal, which is dissipated to the outer root surface. Previous studies ^16^ ^,^ ^28^ show that a temperature increase of 10º C from a body temperature of 37º C can cause irreparable damage to periradicular tissues, such as bone necrosis. Otherwise, a healthy periradicular vascular system and the thermal conductivity of the periodontal ligament, dentin, and alveolar bone help to dissipate high temperatures ^12^. The highest temperature obtained in the study was 43.3º C in the US group, indicating that the methods studied had not caused damage to the support structures when the protocol suggested by the manufacturer was applied. Other researchers investigating thermoplasticizing techniques have found that temperature increases on the root surface are not sufficiently high enough to cause damage to the adjacent periodontal tissues ^29^.

In this study, the irrigant (NaOCI) was used at room temperature, and it was not possible to measure the intracanal temperature due to the nature of the experiment. Another study showed that heating the NaOCI solution enhanced its ability to dissolve organic material ^30^. Heating NaOCl improved their capacity to dissolve organic matter, and collagen, and remove the smear layer ^12^ ^,^ ^15^. It is difficult to maintain a temperature. The solution is constantly agitated and heated. When the agitation stops, the temperature immediately drops.

There is no definitive consensus on the usage time of irrigation agitators; several protocols can be found ^6^ ^,^ ^7^ ^,^ ^10^ ^,^ ^20^. In the present study, 30 seconds were considered for all groups. In the literature, between 30 seconds and 3 minutes are recommended for NaOCl irrigation. However, when the instrument is subjected to a shorter period of passive irrigation, such as 30 seconds, there is a reduction in risks, such as instrument fracture or deformation of the walls ^31^.

In the present study, K-type thermocouple temperature sensors were used to measure the heat generated inside the root canal and transmitted to the external root surface. Different methods have already been used to measure external root temperature, such as thermographic temperature cameras. However, camera calibration can be affected by variables that are difficult to control, such as ambient temperature and distance from the root to the camera ^18^ ^,^ ^32^.

Thermocouples have been the traditional gold standard of temperature measurement ^32^. Can measure temperatures between -200ºC and 1200ºC ^32^ ^,^ ^33^. The use of this type of sensor allows the temperature to be measured in different thirds of the root canal ^20^ ^,^ ^33^, making it the most appropriate resource for this type of experiment. On the other hand, they may have an error limit of ±0.4 Cº to ± 0.75 Cº.

In the present study, a water bath guaranteed a constant temperature of 37°C during all irrigation procedures. This model is similar to experiments focusing on temperature increases (16, 34) but it still has limitations, for example, the lack of simulation of blood flow in the periodontal ligament and alveolar bone ^21^, which can also contribute to heat dissipation and cannot replace a clinical investigation.

The agitation technique using the ultrasonic tip did not exceed the temperature limit but obtained significantly greater temperature changes than the other techniques, especially in the cervical and apical thirds. So, the null hypothesis was rejected. This finding is in accordance with the literature ^20^. Ultrasonic waves occur when particles are energized, causing vibration and energy transfers ^35^ ^,^ ^36^. These waves are converted into mechanical energy through ultrasonic transducers, being converted into heat through ultrasonic activation in the solution during cavitation ^37^.

The heat is transmitted from the ultrasonic source to the ultrasonic tip, and from the ultrasonic tip to the solution, therefore, there is more expressive heating close to the ultrasonic source ^34^. In the cervical third, there is a greater amount of irrigant and greater proximity to the ultrasonic source compared to the middle third, generating heat dissipation in this region. On the other hand, during activation, the instrument may touch the canal walls, generating frictional heat ^16^, as in the apical third, which has a smaller diameter compared to the other thirds, due to the progressive conical configuration after chemomechanical preparation, in addition to the lower amount of irrigant in this area.

No statistical difference was found between the Easyclean instrument and the XP-endo Finisher. Although both have been used in rotary kinematics, it is assumed that the results are associated with the individual characteristics of each instrument. The Easyclean instrument (25.04) is designed for use in reciprocating motion to prevent threading and fracture of the instrument ^38^. However, a recent study was conducted using the instrument in rotary motion (15,000 RPM), and the ability to remove debris was more efficient ^11^. The Easyclean tip touches the root canal walls despite the 04 taper. It is an instrument made of plastic material that does not cut dentin ^39^, can be deformed in the rotational movement, reducing friction with the canal walls, and presents a smaller temperature change of the instruments in rotary kinematics.

The XP-endo Finisher file presented the smallest temperature change between the studied groups. As it is an instrument without a taper (25.00), the use of the system did not promote heating of the external root surface, indicating that the cleaning effectiveness is not only due to the friction of the instrument but also to the flow of fluids in all directions and the irrigant action ^39^.

A limitation of the present study can be associated with the exclusive use of teeth with single roots and straight curvature, according to Schneider ^23^. This same limitation is found in previous articles ^16^ ^,^ ^21^ therefore, it may not reflect the challenges to which these methods of agitation are subject in a clinical situation. Therefore, future research on teeth that present anatomical variations is necessary. Another limitation of the study is the fact that thermocouples do not measure all the external root surfaces but seem to be the most appropriate resource for this kind of experiment ^40^.

In conclusion, none of the agitation methods of the irrigants studied (Irrisonic, XP-endoFinisher, and Easyclean) presented heating above 47ºC, so they can be performed safely without causing damage to the tissues adjacent to the external surface of the dental roots.
